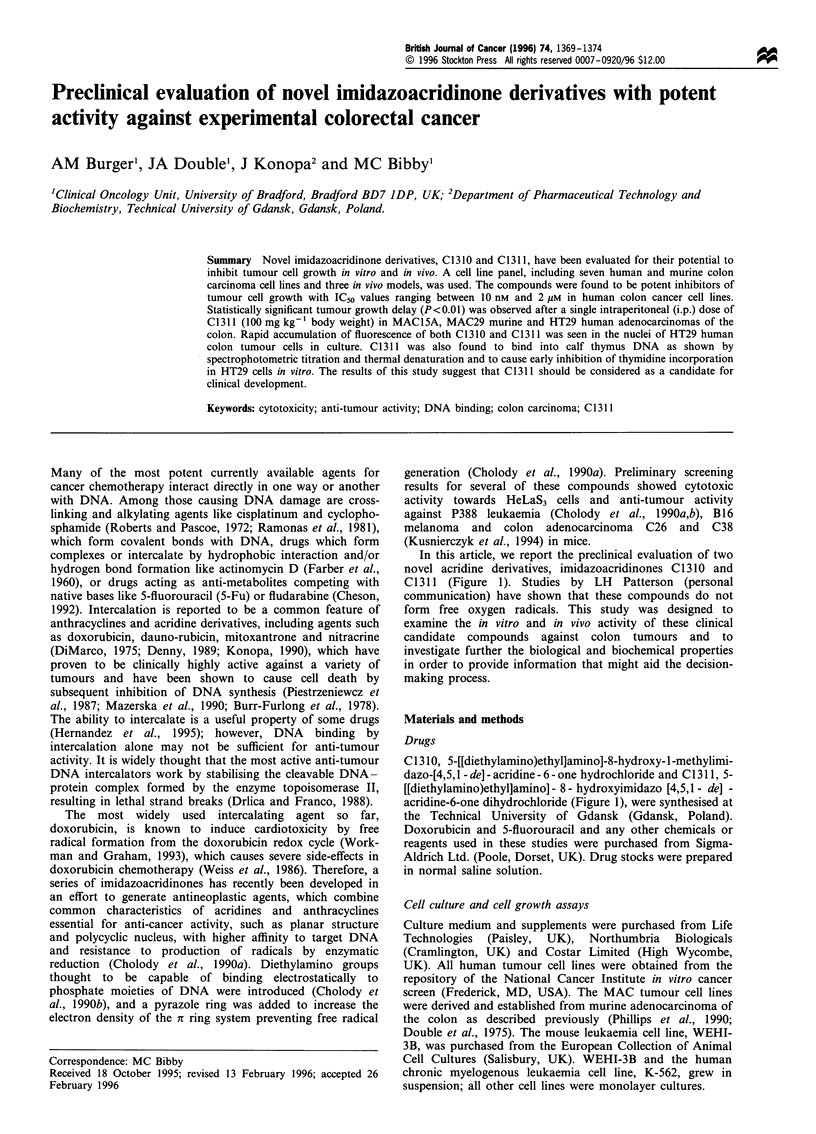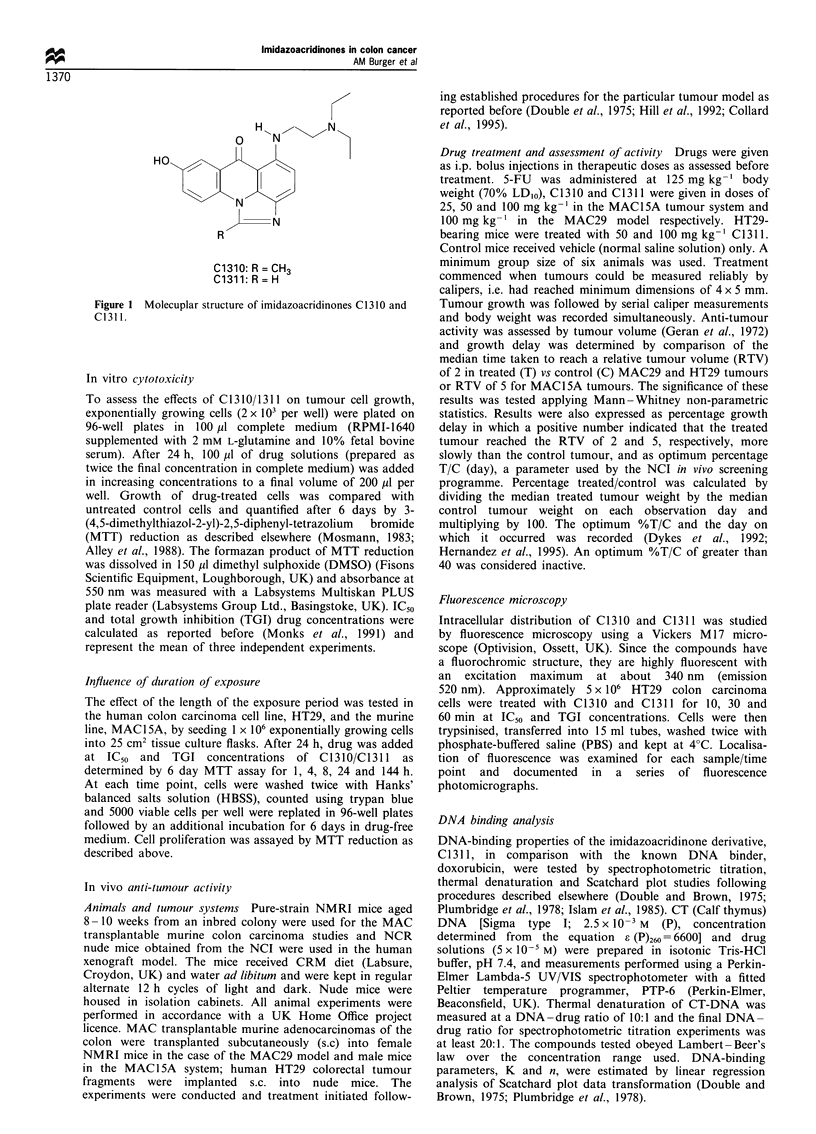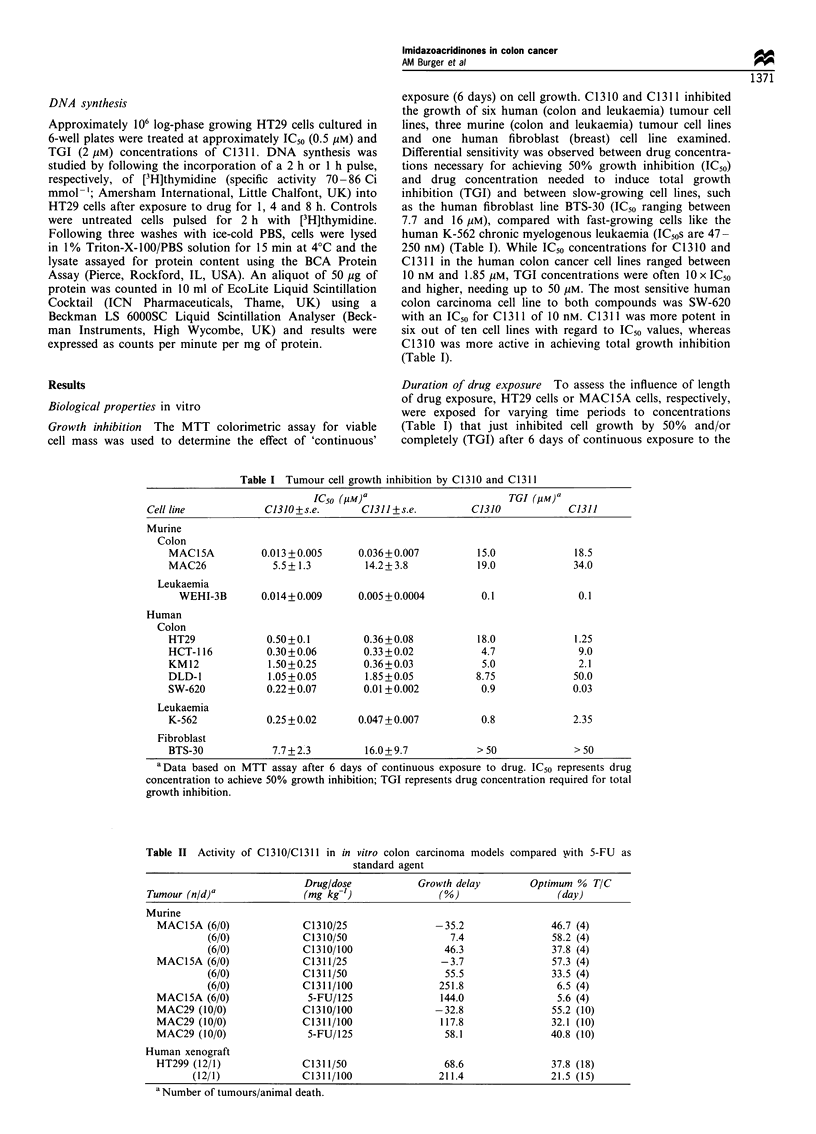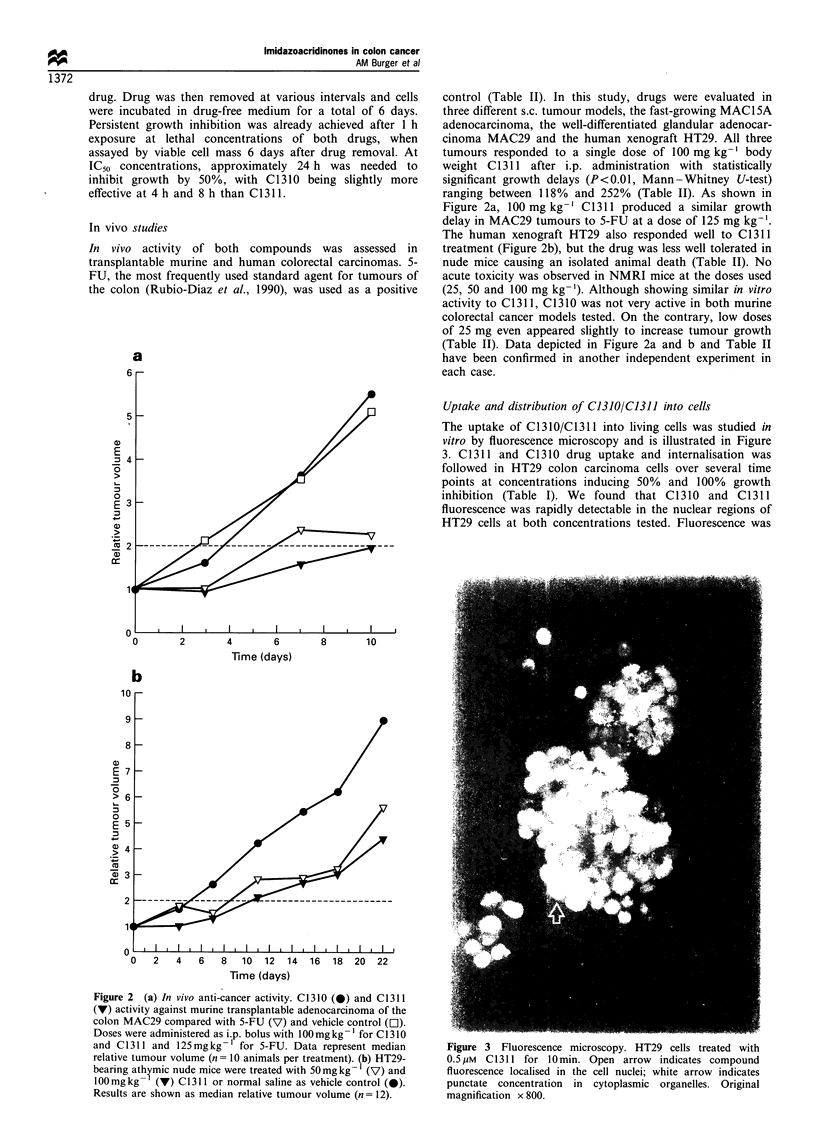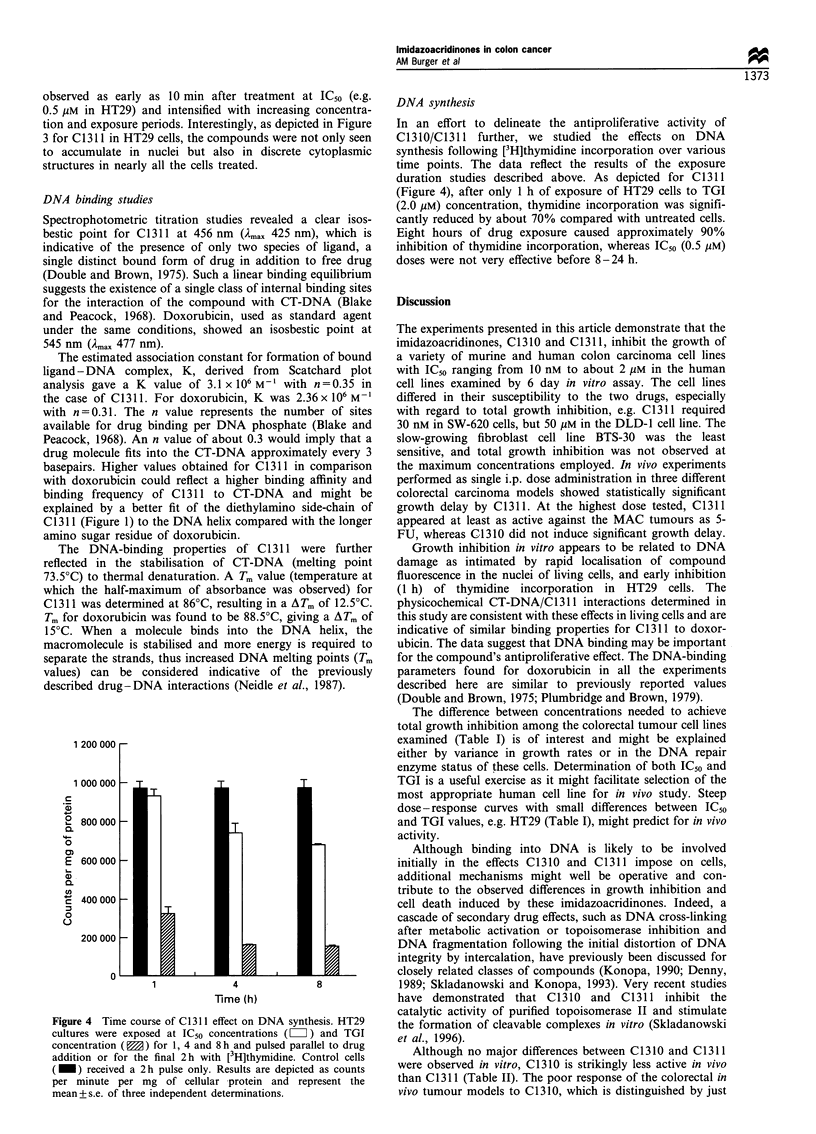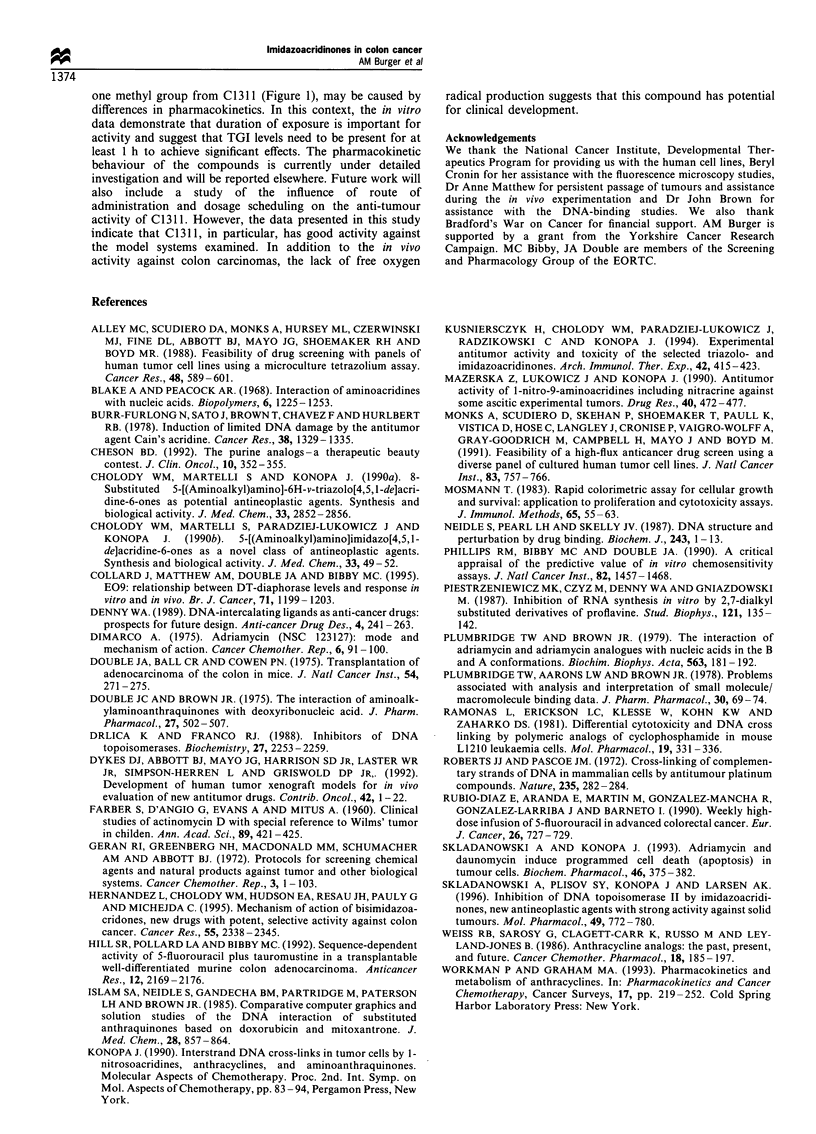# Preclinical evaluation of novel imidazoacridinone derivatives with potent activity against experimental colorectal cancer.

**DOI:** 10.1038/bjc.1996.551

**Published:** 1996-11

**Authors:** A. M. Burger, J. A. Double, J. Konopa, M. C. Bibby

**Affiliations:** Clinical Oncology Unit, University of Bradford, UK.

## Abstract

**Images:**


British Journal of Cancer (1996) 74, 1369-1374
© 1996 Stockton Press All rights reserved 0007-0920/96 $12.00

### Preclinical evaluation of novel imidazoacridinone derivatives with potent activity against experimental colorectal cancer

AM Burger[1], JA Double[1], J Konopa[2] and MC Bibby[1]

[1]*Clinical Oncology Unit, University of Bradford, Bradford BD7 1DP, UK;* [2]*Department of Pharmaceutical Technology and Biochemistry, Technical University of Gdansk, Gdansk, Poland.*

**Summary** Novel imidazoacridinone derivatives, C1310 and C1311, have been evaluated for their potential to inhibit tumour cell growth *in vitro* and *in vivo*. A cell line panel, including seven human and murine colon carcinoma cell lines and three *in vivo* models, was used. The compounds were found to be potent inhibitors of tumour cell growth with $IC_{50}$ values ranging between 10 nM and 2 $\mu$M in human colon cancer cell lines. Statistically significant tumour growth delay ($P<0.01$) was observed after a single intraperitoneal (i.p.) dose of C1311 (100 mg $kg^{-1}$ body weight) in MAC15A, MAC29 murine and HT29 human adenocarcinomas of the colon. Rapid accumulation of fluorescence of both C1310 and C1311 was seen in the nuclei of HT29 human colon tumour cells in culture. C1311 was also found to bind into calf thymus DNA as shown by spectrophotometric titration and thermal denaturation and to cause early inhibition of thymidine incorporation in HT29 cells *in vitro*. The results of this study suggest that C1311 should be considered as a candidate for clinical development.

**Keywords:** cytotoxicity; anti-tumour activity; DNA binding; colon carcinoma; C1311

Many of the most potent currently available agents for cancer chemotherapy interact directly in one way or another with DNA. Among those causing DNA damage are cross-linking and alkylating agents like cisplatinum and cyclophosphamide (Roberts and Pascoe, 1972; Ramonas *et al.*, 1981), which form covalent bonds with DNA, drugs which form complexes or intercalate by hydrophobic interaction and/or hydrogen bond formation like actinomycin D (Farber *et al.*, 1960), or drugs acting as anti-metabolites competing with native bases like 5-fluorouracil (5-Fu) or fludarabine (Cheson, 1992). Intercalation is reported to be a common feature of anthracyclines and acridine derivatives, including agents such as doxorubicin, dauno-rubicin, mitoxantrone and nitracrine (DiMarco, 1975; Denny, 1989; Konopa, 1990), which have proven to be clinically highly active against a variety of tumours and have been shown to cause cell death by subsequent inhibition of DNA synthesis (Piestrzeniewcz *et al.*, 1987; Mazerska *et al.*, 1990; Burr-Furlong *et al.*, 1978). The ability to intercalate is a useful property of some drugs (Hernandez *et al.*, 1995); however, DNA binding by intercalation alone may not be sufficient for anti-tumour activity. It is widely thought that the most active anti-tumour DNA intercalators work by stabilising the cleavable DNA–protein complex formed by the enzyme topoisomerase II, resulting in lethal strand breaks (Drlica and Franco, 1988).

The most widely used intercalating agent so far, doxorubicin, is known to induce cardiotoxicity by free radical formation from the doxorubicin redox cycle (Workman and Graham, 1993), which causes severe side-effects in doxorubicin chemotherapy (Weiss *et al.*, 1986). Therefore, a series of imidazoacridinones has recently been developed in an effort to generate antineoplastic agents, which combine common characteristics of acridines and anthracyclines essential for anti-cancer activity, such as planar structure and polycyclic nucleus, with higher affinity to target DNA and resistance to production of radicals by enzymatic reduction (Cholody *et al.*, 1990*a*). Diethylamino groups thought to be capable of binding electrostatically to phosphate moieties of DNA were introduced (Cholody *et al.*, 1990*b*), and a pyrazole ring was added to increase the electron density of the $\pi$ ring system preventing free radical generation (Cholody *et al.*, 1990*a*). Preliminary screening results for several of these compounds showed cytotoxic activity towards $HeLaS_3$ cells and anti-tumour activity against P388 leukaemia (Cholody *et al.*, 1990*a,b*), B16 melanoma and colon adenocarcinoma C26 and C38 (Kusnierczyk *et al.*, 1994) in mice.

In this article, we report the preclinical evaluation of two novel acridine derivatives, imidazoacridinones C1310 and C1311 (Figure 1). Studies by LH Patterson (personal communication) have shown that these compounds do not form free oxygen radicals. This study was designed to examine the *in vitro* and *in vivo* activity of these clinical candidate compounds against colon tumours and to investigate further the biological and biochemical properties in order to provide information that might aid the decision-making process.

### Materials and methods

### *Drugs*

C1310, 5-[[diethylamino)ethyl]amino]-8-hydroxy-1-methylimidazo-[4,5,1-*de*]-acridine-6-one hydrochloride and C1311, 5-[[diethylamino)ethyl]amino]-8-hydroxyimidazo [4,5,1-*de*]-acridine-6-one dihydrochloride (Figure 1), were synthesised at the Technical University of Gdansk (Gdansk, Poland). Doxorubicin and 5-fluorouracil and any other chemicals or reagents used in these studies were purchased from Sigma-Aldrich Ltd. (Poole, Dorset, UK). Drug stocks were prepared in normal saline solution.

### *Cell culture and cell growth assays*

Culture medium and supplements were purchased from Life Technologies (Paisley, UK), Northumbria Biologicals (Cramlington, UK) and Costar Limited (High Wycombe, UK). All human tumour cell lines were obtained from the repository of the National Cancer Institute *in vitro* cancer screen (Frederick, MD, USA). The MAC tumour cell lines were derived and established from murine adenocarcinoma of the colon as described previously (Phillips *et al.*, 1990; Double *et al.*, 1975). The mouse leukaemia cell line, WEHI-3B, was purchased from the European Collection of Animal Cell Cultures (Salisbury, UK). WEHI-3B and the human chronic myelogenous leukaemia cell line, K-562, grew in suspension; all other cell lines were monolayer cultures.

Correspondence: MC Bibby
Received 18 October 1995; revised 13 February 1996; accepted 26 February 1996

Figure 1 Molecuplar structure of imidazoacridinones C1310 and C1311.

### In vitro *cytotoxicity*

To assess the effects of C1310/1311 on tumour cell growth, exponentially growing cells ($2 \times 10^3$ per well) were plated on 96-well plates in 100 $\mu$l complete medium (RPMI-1640 supplemented with 2 mM L-glutamine and 10% fetal bovine serum). After 24 h, 100 $\mu$l of drug solutions (prepared as twice the final concentration in complete medium) was added in increasing concentrations to a final volume of 200 $\mu$l per well. Growth of drug-treated cells was compared with untreated control cells and quantified after 6 days by 3-(4,5-dimethylthiazol-2-yl)-2,5-diphenyl-tetrazolium bromide (MTT) reduction as described elsewhere (Mosmann, 1983; Alley *et al.*, 1988). The formazan product of MTT reduction was dissolved in 150 $\mu$l dimethyl sulphoxide (DMSO) (Fisons Scientific Equipment, Loughborough, UK) and absorbance at 550 nm was measured with a Labsystems Multiskan PLUS plate reader (Labsystems Group Ltd., Basingstoke, UK). $IC_{50}$ and total growth inhibition (TGI) drug concentrations were calculated as reported before (Monks *et al.*, 1991) and represent the mean of three independent experiments.

### *Influence of duration of exposure*

The effect of the length of the exposure period was tested in the human colon carcinoma cell line, HT29, and the murine line, MAC15A, by seeding $1 \times 10^6$ exponentially growing cells into 25 $cm^2$ tissue culture flasks. After 24 h, drug was added at $IC_{50}$ and TGI concentrations of C1310/C1311 as determined by 6 day MTT assay for 1, 4, 8, 24 and 144 h. At each time point, cells were washed twice with Hanks' balanced salts solution (HBSS), counted using trypan blue and 5000 viable cells per well were replated in 96-well plates followed by an additional incubation for 6 days in drug-free medium. Cell proliferation was assayed by MTT reduction as described above.

### In vivo *anti-tumour activity*

*Animals and tumour systems* Pure-strain NMRI mice aged 8–10 weeks from an inbred colony were used for the MAC transplantable murine colon carcinoma studies and NCR nude mice obtained from the NCI were used in the human xenograft model. The mice received CRM diet (Labsure, Croydon, UK) and water *ad libitum* and were kept in regular alternate 12 h cycles of light and dark. Nude mice were housed in isolation cabinets. All animal experiments were performed in accordance with a UK Home Office project licence. MAC transplantable murine adenocarcinomas of the colon were transplanted subcutaneously (s.c) into female NMRI mice in the case of the MAC29 model and male mice in the MAC15A system; human HT29 colorectal tumour fragments were implanted s.c. into nude mice. The experiments were conducted and treatment initiated following established procedures for the particular tumour model as reported before (Double *et al.*, 1975; Hill *et al.*, 1992; Collard *et al.*, 1995).

*Drug treatment and assessment of activity* Drugs were given as i.p. bolus injections in therapeutic doses as assessed before treatment. 5-FU was administered at 125 mg $kg^{-1}$ body weight (70% $LD_{10}$), C1310 and C1311 were given in doses of 25, 50 and 100 mg $kg^{-1}$ in the MAC15A tumour system and 100 mg $kg^{-1}$ in the MAC29 model respectively. HT29-bearing mice were treated with 50 and 100 mg $kg^{-1}$ C1311. Control mice received vehicle (normal saline solution) only. A minimum group size of six animals was used. Treatment commenced when tumours could be measured reliably by calipers, i.e. had reached minimum dimensions of $4 \times 5$ mm. Tumour growth was followed by serial caliper measurements and body weight was recorded simultaneously. Anti-tumour activity was assessed by tumour volume (Geran *et al.*, 1972) and growth delay was determined by comparison of the median time taken to reach a relative tumour volume (RTV) of 2 in treated (T) *vs* control (C) MAC29 and HT29 tumours or RTV of 5 for MAC15A tumours. The significance of these results was tested applying Mann–Whitney non-parametric statistics. Results were also expressed as percentage growth delay in which a positive number indicated that the treated tumour reached the RTV of 2 and 5, respectively, more slowly than the control tumour, and as optimum percentage T/C (day), a parameter used by the NCI *in vivo* screening programme. Percentage treated/control was calculated by dividing the median treated tumour weight by the median control tumour weight on each observation day and multiplying by 100. The optimum %T/C and the day on which it occurred was recorded (Dykes *et al.*, 1992; Hernandez *et al.*, 1995). An optimum %T/C of greater than 40 was considered inactive.

### *Fluorescence microscopy*

Intracellular distribution of C1310 and C1311 was studied by fluorescence microscopy using a Vickers M17 microscope (Optivision, Ossett, UK). Since the compounds have a fluorochromic structure, they are highly fluorescent with an excitation maximum at about 340 nm (emission 520 nm). Approximately $5 \times 10^6$ HT29 colon carcinoma cells were treated with C1310 and C1311 for 10, 30 and 60 min at $IC_{50}$ and TGI concentrations. Cells were then trypsinised, transferred into 15 ml tubes, washed twice with phosphate-buffered saline (PBS) and kept at 4°C. Localisation of fluorescence was examined for each sample/time point and documented in a series of fluorescence photomicrographs.

### *DNA binding analysis*

DNA-binding properties of the imidazoacridinone derivative, C1311, in comparison with the known DNA binder, doxorubicin, were tested by spectrophotometric titration, thermal denaturation and Scatchard plot studies following procedures described elsewhere (Double and Brown, 1975; Plumbridge *et al.*, 1978; Islam *et al.*, 1985). CT (Calf thymus) DNA [Sigma type I; $2.5 \times 10^{-3}$ M (P), concentration determined from the equation $\varepsilon (P)_{260} = 6600$] and drug solutions ($5 \times 10^{-5}$ M) were prepared in isotonic Tris-HCl buffer, pH 7.4, and measurements performed using a Perkin-Elmer Lambda-5 UV/VIS spectrophotometer with a fitted Peltier temperature programmer, PTP-6 (Perkin-Elmer, Beaconsfield, UK). Thermal denaturation of CT-DNA was measured at a DNA–drug ratio of 10:1 and the final DNA–drug ratio for spectrophotometric titration experiments was at least 20:1. The compounds tested obeyed Lambert–Beer's law over the concentration range used. DNA-binding parameters, K and *n*, were estimated by linear regression analysis of Scatchard plot data transformation (Double and Brown, 1975; Plumbridge *et al.*, 1978).

*DNA synthesis*

Approximately $10^6$ log-phase growing HT29 cells cultured in 6-well plates were treated at approximately $IC_{50}$ (0.5 $\mu$M) and TGI (2 $\mu$M) concentrations of C1311. DNA synthesis was studied by following the incorporation of a 2 h or 1 h pulse, respectively, of [$^3$H]thymidine (specific activity 70–86 Ci mmol$^{-1}$; Amersham International, Little Chalfont, UK) into HT29 cells after exposure to drug for 1, 4 and 8 h. Controls were untreated cells pulsed for 2 h with [$^3$H]thymidine. Following three washes with ice-cold PBS, cells were lysed in 1% Triton-X-100/PBS solution for 15 min at 4°C and the lysate assayed for protein content using the BCA Protein Assay (Pierce, Rockford, IL, USA). An aliquot of 50 $\mu$g of protein was counted in 10 ml of EcoLite Liquid Scintillation Cocktail (ICN Pharmaceuticals, Thame, UK) using a Beckman LS 6000SC Liquid Scintillation Analyser (Beckman Instruments, High Wycombe, UK) and results were expressed as counts per minute per mg of protein.

### Results

*Biological properties* in vitro

*Growth inhibition* The MTT colorimetric assay for viable cell mass was used to determine the effect of 'continuous' exposure (6 days) on cell growth. C1310 and C1311 inhibited the growth of six human (colon and leukaemia) tumour cell lines, three murine (colon and leukaemia) tumour cell lines and one human fibroblast (breast) cell line examined. Differential sensitivity was observed between drug concentrations necessary for achieving 50% growth inhibition ($IC_{50}$) and drug concentration needed to induce total growth inhibition (TGI) and between slow-growing cell lines, such as the human fibroblast line BTS-30 ($IC_{50}$ ranging between 7.7 and 16 $\mu$M), compared with fast-growing cells like the human K-562 chronic myelogenous leukaemia ($IC_{50}$s are 47–250 nM) (Table I). While $IC_{50}$ concentrations for C1310 and C1311 in the human colon cancer cell lines ranged between 10 nM and 1.85 $\mu$M, TGI concentrations were often $10 \times IC_{50}$ and higher, needing up to 50 $\mu$M. The most sensitive human colon carcinoma cell line to both compounds was SW-620 with an $IC_{50}$ for C1311 of 10 nM. C1311 was more potent in six out of ten cell lines with regard to $IC_{50}$ values, whereas C1310 was more active in achieving total growth inhibition (Table I).

*Duration of drug exposure* To assess the influence of length of drug exposure, HT29 cells or MAC15A cells, respectively, were exposed for varying time periods to concentrations (Table I) that just inhibited cell growth by 50% and/or completely (TGI) after 6 days of continuous exposure to the

**Table I** Tumour cell growth inhibition by C1310 and C1311

| Cell line | $IC_{50}$ ($\mu$M)[a] C1310±s.e. | C1311±s.e. | TGI ($\mu$M)[a] C1310 | C1311 |
|---|---|---|---|---|
| Murine | | | | |
| Colon | | | | |
| MAC15A | 0.013±0.005 | 0.036±0.007 | 15.0 | 18.5 |
| MAC26 | 5.5±1.3 | 14.2±3.8 | 19.0 | 34.0 |
| Leukaemia | | | | |
| WEHI-3B | 0.014±0.009 | 0.005±0.0004 | 0.1 | 0.1 |
| Human | | | | |
| Colon | | | | |
| HT29 | 0.50±0.1 | 0.36±0.08 | 18.0 | 1.25 |
| HCT-116 | 0.30±0.06 | 0.33±0.02 | 4.7 | 9.0 |
| KM12 | 1.50±0.25 | 0.36±0.03 | 5.0 | 2.1 |
| DLD-1 | 1.05±0.05 | 1.85±0.05 | 8.75 | 50.0 |
| SW-620 | 0.22±0.07 | 0.01±0.002 | 0.9 | 0.03 |
| Leukaemia | | | | |
| K-562 | 0.25±0.02 | 0.047±0.007 | 0.8 | 2.35 |
| Fibroblast | | | | |
| BTS-30 | 7.7±2.3 | 16.0±9.7 | >50 | >50 |

[a] Data based on MTT assay after 6 days of continuous exposure to drug. $IC_{50}$ represents drug concentration to achieve 50% growth inhibition; TGI represents drug concentration required for total growth inhibition.

**Table II** Activity of C1310/C1311 in *in vitro* colon carcinoma models compared with 5-FU as standard agent

| Tumour (n/d)[a] | Drug/dose (mg kg$^{-1}$) | Growth delay (%) | Optimum % T/C (day) |
|---|---|---|---|
| Murine | | | |
| MAC15A (6/0) | C1310/25 | −35.2 | 46.7 (4) |
| (6/0) | C1310/50 | 7.4 | 58.2 (4) |
| (6/0) | C1310/100 | 46.3 | 37.8 (4) |
| MAC15A (6/0) | C1311/25 | −3.7 | 57.3 (4) |
| (6/0) | C1311/50 | 55.5 | 33.5 (4) |
| (6/0) | C1311/100 | 251.8 | 6.5 (4) |
| MAC15A (6/0) | 5-FU/125 | 144.0 | 5.6 (4) |
| MAC29 (10/0) | C1310/100 | −32.8 | 55.2 (10) |
| MAC29 (10/0) | C1311/100 | 117.8 | 32.1 (10) |
| MAC29 (10/0) | 5-FU/125 | 58.1 | 40.8 (10) |
| Human xenograft | | | |
| HT299 (12/1) | C1311/50 | 68.6 | 37.8 (18) |
| (12/1) | C1311/100 | 211.4 | 21.5 (15) |

[a] Number of tumours/animal death.

drug. Drug was then removed at various intervals and cells were incubated in drug-free medium for a total of 6 days. Persistent growth inhibition was already achieved after 1 h exposure at lethal concentrations of both drugs, when assayed by viable cell mass 6 days after drug removal. At $IC_{50}$ concentrations, approximately 24 h was needed to inhibit growth by 50%, with C1310 being slightly more effective at 4 h and 8 h than C1311.

### In vivo *studies*

*In vivo* activity of both compounds was assessed in transplantable murine and human colorectal carcinomas. 5-FU, the most frequently used standard agent for tumours of the colon (Rubio-Diaz *et al.*, 1990), was used as a positive control (Table II). In this study, drugs were evaluated in three different s.c. tumour models, the fast-growing MAC15A adenocarcinoma, the well-differentiated glandular adenocarcinoma MAC29 and the human xenograft HT29. All three tumours responded to a single dose of 100 mg $kg^{-1}$ body weight C1311 after i.p. administration with statistically significant growth delays ($P<0.01$, Mann–Whitney *U*-test) ranging between 118% and 252% (Table II). As shown in Figure 2a, 100 mg $kg^{-1}$ C1311 produced a similar growth delay in MAC29 tumours to 5-FU at a dose of 125 mg $kg^{-1}$. The human xenograft HT29 also responded well to C1311 treatment (Figure 2b), but the drug was less well tolerated in nude mice causing an isolated animal death (Table II). No acute toxicity was observed in NMRI mice at the doses used (25, 50 and 100 mg $kg^{-1}$). Although showing similar *in vitro* activity to C1311, C1310 was not very active in both murine colorectal cancer models tested. On the contrary, low doses of 25 mg even appeared slightly to increase tumour growth (Table II). Data depicted in Figure 2a and b and Table II have been confirmed in another independent experiment in each case.

### *Uptake and distribution of C1310/C1311 into cells*

The uptake of C1310/C1311 into living cells was studied *in vitro* by fluorescence microscopy and is illustrated in Figure 3. C1311 and C1310 drug uptake and internalisation was followed in HT29 colon carcinoma cells over several time points at concentrations inducing 50% and 100% growth inhibition (Table I). We found that C1310 and C1311 fluorescence was rapidly detectable in the nuclear regions of HT29 cells at both concentrations tested. Fluorescence was

**Figure 2** (**a**) *In vivo* anti-cancer activity. C1310 (●) and C1311 (▼) activity against murine transplantable adenocarcinoma of the colon MAC29 compared with 5-FU (▽) and vehicle control (□). Doses were administered as i.p. bolus with 100 mg $kg^{-1}$ for C1310 and C1311 and 125 mg $kg^{-1}$ for 5-FU. Data represent median relative tumour volume ($n=10$ animals per treatment). (**b**) HT29-bearing athymic nude mice were treated with 50 mg $kg^{-1}$ (▽) and 100 mg $kg^{-1}$ (▼) C1311 or normal saline as vehicle control (●). Results are shown as median relative tumour volume ($n=12$).

**Figure 3** Fluorescence microscopy. HT29 cells treated with 0.5 $\mu$M C1311 for 10 min. Open arrow indicates compound fluorescence localised in the cell nuclei; white arrow indicates punctate concentration in cytoplasmic organelles. Original magnification ×800.

observed as early as 10 min after treatment at $IC_{50}$ (e.g. 0.5 $\mu$M in HT29) and intensified with increasing concentration and exposure periods. Interestingly, as depicted in Figure 3 for C1311 in HT29 cells, the compounds were not only seen to accumulate in nuclei but also in discrete cytoplasmic structures in nearly all the cells treated.

*DNA binding studies*

Spectrophotometric titration studies revealed a clear isosbestic point for C1311 at 456 nm ($\lambda_{max}$ 425 nm), which is indicative of the presence of only two species of ligand, a single distinct bound form of drug in addition to free drug (Double and Brown, 1975). Such a linear binding equilibrium suggests the existence of a single class of internal binding sites for the interaction of the compound with CT-DNA (Blake and Peacock, 1968). Doxorubicin, used as standard agent under the same conditions, showed an isosbestic point at 545 nm ($\lambda_{max}$ 477 nm).

The estimated association constant for formation of bound ligand–DNA complex, K, derived from Scatchard plot analysis gave a K value of $3.1 \times 10^6$ $M^{-1}$ with $n = 0.35$ in the case of C1311. For doxorubicin, K was $2.36 \times 10^6$ $M^{-1}$ with $n = 0.31$. The $n$ value represents the number of sites available for drug binding per DNA phosphate (Blake and Peacock, 1968). An $n$ value of about 0.3 would imply that a drug molecule fits into the CT-DNA approximately every 3 basepairs. Higher values obtained for C1311 in comparison with doxorubicin could reflect a higher binding affinity and binding frequency of C1311 to CT-DNA and might be explained by a better fit of the diethylamino side-chain of C1311 (Figure 1) to the DNA helix compared with the longer amino sugar residue of doxorubicin.

The DNA-binding properties of C1311 were further reflected in the stabilisation of CT-DNA (melting point 73.5°C) to thermal denaturation. A $T_m$ value (temperature at which the half-maximum of absorbance was observed) for C1311 was determined at 86°C, resulting in a $\Delta T_m$ of 12.5°C. $T_m$ for doxorubicin was found to be 88.5°C, giving a $\Delta T_m$ of 15°C. When a molecule binds into the DNA helix, the macromolecule is stabilised and more energy is required to separate the strands, thus increased DNA melting points ($T_m$ values) can be considered indicative of the previously described drug–DNA interactions (Neidle *et al.*, 1987).

**Figure 4** Time course of C1311 effect on DNA synthesis. HT29 cultures were exposed at $IC_{50}$ concentrations (□) and TGI concentration (▨) for 1, 4 and 8 h and pulsed parallel to drug addition or for the final 2 h with [$^3$H]thymidine. Control cells (■) received a 2 h pulse only. Results are depicted as counts per minute per mg of cellular protein and represent the mean ± s.e. of three independent determinations.

*DNA synthesis*

In an effort to delineate the antiproliferative activity of C1310/C1311 further, we studied the effects on DNA synthesis following [$^3$H]thymidine incorporation over various time points. The data reflect the results of the exposure duration studies described above. As depicted for C1311 (Figure 4), after only 1 h of exposure of HT29 cells to TGI (2.0 $\mu$M) concentration, thymidine incorporation was significantly reduced by about 70% compared with untreated cells. Eight hours of drug exposure caused approximately 90% inhibition of thymidine incorporation, whereas $IC_{50}$ (0.5 $\mu$M) doses were not very effective before 8–24 h.

### Discussion

The experiments presented in this article demonstrate that the imidazoacridinones, C1310 and C1311, inhibit the growth of a variety of murine and human colon carcinoma cell lines with $IC_{50}$ ranging from 10 nM to about 2 $\mu$M in the human cell lines examined by 6 day *in vitro* assay. The cell lines differed in their susceptibility to the two drugs, especially with regard to total growth inhibition, e.g. C1311 required 30 nM in SW-620 cells, but 50 $\mu$M in the DLD-1 cell line. The slow-growing fibroblast cell line BTS-30 was the least sensitive, and total growth inhibition was not observed at the maximum concentrations employed. *In vivo* experiments performed as single i.p. dose administration in three different colorectal carcinoma models showed statistically significant growth delay by C1311. At the highest dose tested, C1311 appeared at least as active against the MAC tumours as 5-FU, whereas C1310 did not induce significant growth delay.

Growth inhibition *in vitro* appears to be related to DNA damage as intimated by rapid localisation of compound fluorescence in the nuclei of living cells, and early inhibition (1 h) of thymidine incorporation in HT29 cells. The physicochemical CT-DNA/C1311 interactions determined in this study are consistent with these effects in living cells and are indicative of similar binding properties for C1311 to doxorubicin. The data suggest that DNA binding may be important for the compound's antiproliferative effect. The DNA-binding parameters found for doxorubicin in all the experiments described here are similar to previously reported values (Double and Brown, 1975; Plumbridge and Brown, 1979).

The difference between concentrations needed to achieve total growth inhibition among the colorectal tumour cell lines examined (Table I) is of interest and might be explained either by variance in growth rates or in the DNA repair enzyme status of these cells. Determination of both $IC_{50}$ and TGI is a useful exercise as it might facilitate selection of the most appropriate human cell line for *in vivo* study. Steep dose–response curves with small differences between $IC_{50}$ and TGI values, e.g. HT29 (Table I), might predict for *in vivo* activity.

Although binding into DNA is likely to be involved initially in the effects C1310 and C1311 impose on cells, additional mechanisms might well be operative and contribute to the observed differences in growth inhibition and cell death induced by these imidazoacridinones. Indeed, a cascade of secondary drug effects, such as DNA cross-linking after metabolic activation or topoisomerase inhibition and DNA fragmentation following the initial distortion of DNA integrity by intercalation, have previously been discussed for closely related classes of compounds (Konopa, 1990; Denny, 1989; Skladanowski and Konopa, 1993). Very recent studies have demonstrated that C1310 and C1311 inhibit the catalytic activity of purified topoisomerase II and stimulate the formation of cleavable complexes *in vitro* (Skladanowski *et al.*, 1996).

Although no major differences between C1310 and C1311 were observed *in vitro*, C1310 is strikingly less active *in vivo* than C1311 (Table II). The poor response of the colorectal *in vivo* tumour models to C1310, which is distinguished by just one methyl group from C1311 (Figure 1), may be caused by differences in pharmacokinetics. In this context, the *in vitro* data demonstrate that duration of exposure is important for activity and suggest that TGI levels need to be present for at least 1 h to achieve significant effects. The pharmacokinetic behaviour of the compounds is currently under detailed investigation and will be reported elsewhere. Future work will also include a study of the influence of route of administration and dosage scheduling on the anti-tumour activity of C1311. However, the data presented in this study indicate that C1311, in particular, has good activity against the model systems examined. In addition to the *in vivo* activity against colon carcinomas, the lack of free oxygen radical production suggests that this compound has potential for clinical development.

**Acknowledgements**

We thank the National Cancer Institute, Developmental Therapeutics Program for providing us with the human cell lines, Beryl Cronin for her assistance with the fluorescence microscopy studies, Dr Anne Matthew for persistent passage of tumours and assistance during the *in vivo* experimentation and Dr John Brown for assistance with the DNA-binding studies. We also thank Bradford's War on Cancer for financial support. AM Burger is supported by a grant from the Yorkshire Cancer Research Campaign. MC Bibby, JA Double are members of the Screening and Pharmacology Group of the EORTC.

### References

ALLEY MC, SCUDIERO DA, MONKS A, HURSEY ML, CZERWINSKI MJ, FINE DL, ABBOTT BJ, MAYO JG, SHOEMAKER RH AND BOYD MR. (1988). Feasibility of drug screening with panels of human tumor cell lines using a microculture tetrazolium assay. *Cancer Res.*, **48**, 589–601.

BLAKE A AND PEACOCK AR. (1968). Interaction of aminoacridines with nucleic acids. *Biopolymers*, **6**, 1225–1253.

BURR-FURLONG N, SATO J, BROWN T, CHAVEZ F AND HURLBERT RB. (1978). Induction of limited DNA damage by the antitumor agent Cain's acridine. *Cancer Res.*, **38**, 1329–1335.

CHESON BD. (1992). The purine analogs–a therapeutic beauty contest. *J. Clin. Oncol.*, **10**, 352–355.

CHOLODY WM, MARTELLI S AND KONOPA J. (1990*a*). 8-Substituted 5-[(Aminoalkyl)amino]-6H-*v*-triazolo[4,5,1-*de*]acridine-6-ones as potential antineoplastic agents. Synthesis and biological activity. *J. Med. Chem.*, **33**, 2852–2856.

CHOLODY WM, MARTELLI S, PARADZIEJ-LUKOWICZ J AND KONOPA J. (1990*b*). 5-[(Aminoalkyl)amino]imidazo[4,5,1-*de*]acridine-6-ones as a novel class of antineoplastic agents. Synthesis and biological activity. *J. Med. Chem.*, **33**, 49–52.

COLLARD J, MATTHEW AM, DOUBLE JA AND BIBBY MC. (1995). EO9: relationship between DT-diaphorase levels and response *in vitro* and *in vivo*. *Br. J. Cancer*, **71**, 1199–1203.

DENNY WA. (1989). DNA-intercalating ligands as anti-cancer drugs: prospects for future design. *Anti-cancer Drug Des.*, **4**, 241–263.

DIMARCO A. (1975). Adriamycin (NSC 123127): mode and mechanism of action. *Cancer Chemother. Rep.*, **6**, 91–100.

DOUBLE JA, BALL CR AND COWEN PN. (1975). Transplantation of adenocarcinoma of the colon in mice. *J. Natl Cancer Inst.*, **54**, 271–275.

DOUBLE JC AND BROWN JR. (1975). The interaction of aminoalkylaminoanthraquinones with deoxyribonucleic acid. *J. Pharm. Pharmacol.*, **27**, 502–507.

DRLICA K AND FRANCO RJ. (1988). Inhibitors of DNA topoisomerases. *Biochemistry*, **27**, 2253–2259.

DYKES DJ, ABBOTT BJ, MAYO JG, HARRISON SD JR, LASTER WR JR, SIMPSON-HERREN L AND GRISWOLD DP JR,. (1992). Development of human tumor xenograft models for *in vivo* evaluation of new antitumor drugs. *Contrib. Oncol.*, **42**, 1–22.

FARBER S, D'ANGIO G, EVANS A AND MITUS A. (1960). Clinical studies of actinomycin D with special reference to Wilms' tumor in childen. *Ann. Acad. Sci.*, **89**, 421–425.

GERAN RI, GREENBERG NH, MACDONALD MM, SCHUMACHER AM AND ABBOTT BJ. (1972). Protocols for screening chemical agents and natural products against tumor and other biological systems. *Cancer Chemother. Rep.*, **3**, 1–103.

HERNANDEZ L, CHOLODY WM, HUDSON EA, RESAU JH, PAULY G AND MICHEJDA C. (1995). Mechanism of action of bisimidazoacridones, new drugs with potent, selective activity against colon cancer. *Cancer Res.*, **55**, 2338–2345.

HILL SR, POLLARD LA AND BIBBY MC. (1992). Sequence-dependent activity of 5-fluorouracil plus tauromustine in a transplantable well-differentiated murine colon adenocarcinoma. *Anticancer Res.*, **12**, 2169–2176.

ISLAM SA, NEIDLE S, GANDECHA BM, PARTRIDGE M, PATERSON LH AND BROWN JR. (1985). Comparative computer graphics and solution studies of the DNA interaction of substituted anthraquinones based on doxorubicin and mitoxantrone. *J. Med. Chem.*, **28**, 857–864.

KONOPA J. (1990). Interstrand DNA cross-links in tumor cells by 1-nitrosoacridines, anthracyclines, and aminoanthraquinones. Molecular Aspects of Chemotherapy. Proc. 2nd. Int. Symp. on Mol. Aspects of Chemotherapy, pp. 83–94, Pergamon Press, New York.

KUSNIERSCZYK H, CHOLODY WM, PARADZIEJ-LUKOWICZ J, RADZIKOWSKI C AND KONOPA J. (1994). Experimental antitumor activity and toxicity of the selected triazolo- and imidazoacridinones. *Arch. Immunol. Ther. Exp.*, **42**, 415–423.

MAZERSKA Z, LUKOWICZ J AND KONOPA J. (1990). Antitumor activity of 1-nitro-9-aminoacridines including nitracrine against some ascitic experimental tumors. *Drug Res.*, **40**, 472–477.

MONKS A, SCUDIERO D, SKEHAN P, SHOEMAKER T, PAULL K, VISTICA D, HOSE C, LANGLEY J, CRONISE P, VAIGRO-WOLFF A, GRAY-GOODRICH M, CAMPBELL H, MAYO J AND BOYD M. (1991). Feasibility of a high-flux anticancer drug screen using a diverse panel of cultured human tumor cell lines. *J. Natl Cancer Inst.*, **83**, 757–766.

MOSMANN T. (1983). Rapid colorimetric assay for cellular growth and survival: application to proliferation and cytotoxicity assays. *J. Immunol. Methods*, **65**, 55–63.

NEIDLE S, PEARL LH AND SKELLY JV. (1987). DNA structure and perturbation by drug binding. *Biochem. J.*, **243**, 1–13.

PHILLIPS RM, BIBBY MC AND DOUBLE JA. (1990). A critical appraisal of the predictive value of *in vitro* chemosensitivity assays. *J. Natl Cancer Inst.*, **82**, 1457–1468.

PIESTRZENIEWICZ MK, CZYZ M, DENNY WA AND GNIAZDOWSKI M. (1987). Inhibition of RNA synthesis *in vitro* by 2,7-dialkyl substituted derivatives of proflavine. *Stud. Biophys.*, **121**, 135–142.

PLUMBRIDGE TW AND BROWN JR. (1979). The interaction of adriamycin and adriamycin analogues with nucleic acids in the B and A conformations. *Biochim. Biophys. Acta*, **563**, 181–192.

PLUMBRIDGE TW, AARONS LW AND BROWN JR. (1978). Problems associated with analysis and interpretation of small molecule/macromolecule binding data. *J. Pharm. Pharmacol.*, **30**, 69–74.

RAMONAS L, ERICKSON LC, KLESSE W, KOHN KW AND ZAHARKO DS. (1981). Differential cytotoxicity and DNA cross linking by polymeric analogs of cyclophosphamide in mouse L1210 leukaemia cells. *Mol. Pharmacol.*, **19**, 331–336.

ROBERTS JJ AND PASCOE JM. (1972). Cross-linking of complementary strands of DNA in mammalian cells by antitumour platinum compounds. *Nature*, **235**, 282–284.

RUBIO-DIAZ E, ARANDA E, MARTIN M, GONZALEZ-MANCHA R, GONZALEZ-LARRIBA J AND BARNETO I. (1990). Weekly high-dose infusion of 5-fluorouracil in advanced colorectal cancer. *Eur. J. Cancer*, **26**, 727–729.

SKLADANOWSKI A AND KONOPA J. (1993). Adriamycin and daunomycin induce programmed cell death (apoptosis) in tumour cells. *Biochem. Pharmacol.*, **46**, 375–382.

SKLADANOWSKI A, PLISOV SY, KONOPA J AND LARSEN AK. (1996). Inhibition of DNA topoisomerase II by imidazoacridinones, new antineoplastic agents with strong activity against solid tumours. *Mol. Pharmacol.*, **49**, 772–780.

WEISS RB, SAROSY G, CLAGETT-CARR K, RUSSO M AND LEYLAND-JONES B. (1986). Anthracycline analogs: the past, present, and future. *Cancer Chemother. Pharmacol.*, **18**, 185–197.

WORKMAN P AND GRAHAM MA. (1993). Pharmacokinetics and metabolism of anthracyclines. In: *Pharmacokinetics and Cancer Chemotherapy*, Cancer Surveys, **17**, pp. 219–252. Cold Spring Harbor Laboratory Press: New York.